# Oleum Tiglii—Dose Extraordinary

**Published:** 1848-05

**Authors:** J. B. Pride

**Affiliations:** Buffalo


ART. III.—*Oleum Tiglii—Dose Extraodinary.* By J. B. PRIDE, M. D.

On the evening of the seventh inst. I was hastily summoned to visit P. P. R——, a law student in this city, who had, by mistake, twenty-five minutes before, taken a spoonful of Croton oil. Having a slight cold, and attendant cough, which harrassed him somewhat, his mother had administered, as she supposed, a teaspoonful of syrup of Ipecac. Soon after taking the medicine he experienced a very unusual burning sensation in his throat, which induced him to enquire what had been given him ; when, upon examination of the bottle whence the dose taken was poured, it was found that she had administered croton oil, instead of the syrup which she had intended.

I immediately ordered mucilage of Gum Arabic, and directed the patient to drink of it freely, concluding—hastily to be sure, but upon more mature reflection, I think correctly—that by giving freely of mucilage, thus shielding the mucous membrane as effectually as possible, I should do all that could be dône, to prevent the evil that might be expected to result from the passage of so powerful an agent through the alimentary canal.

There were no unnatural symptoms presented, in this case, during the first hour after the oil was taken, except the burning in the fauces, which extended to the œsophagus and stomach, and a general sensation of debility. One hour from the time he took the oil he vomited very freely, ejecting, no doubt, the entire contents of the stomach. I was unable to detect, however, any traces of the oil in the ejected matter.

In two hours from the time the oil was given, it operated very powerfully as a cathartic. The alvine discharges were frequent, indeed almost continuous, for about forty-five minutes, the latter part of the time the evacuations were principally serous, emitting no odor whatever.

Having sustained the system during this violent cathartic operation with diffusible stimulants, I now gave him Sulph. Morph. grs. ss, and prepared more powders containing each ¼ gr. of the same, and directed them to be given once in two hours, if the patient did not sleep—and left him until morning.

8th, 7 o'clock, A. M. Found the patient comfortable ; has slept most of the time since I left him, it having been five hours. The alvine discharges were nearly arrested. Continued the anodyne powders for the next twelve hours. Enjoined rest and abstinence from stimulating food and drink, and continued the mucilage.

7 o'clock, P. M. Has rested quite well most of the day, but for the last two hours has been much annoyed by a painful tenesmus. Ordered anodyne clyster of starch and laudanum, which, in two hours quite overcome this difficulty, having been twice used. Directed him to take of olive oil a teaspoonful once in four hours.

9th, 8 o'clock, A. M. The patient has slept well through the night, and feels very well, but is extremely weak. Continued the olive oil.

8 o'clock, P. M. Had spent the day very pleasantly ; appetite partially restored, moderately indulged, and no aggravation of symptoms. The olive oil, as was intended, has acted as a gentle aperient, producing an almost natural healthy stool. Recommended the continuation of the olive oil occasionally, and suspended treatment in the case. The patient after having suffered *much* from *excessive* irritation, both in the alimentary and urinary passages, and from extreme prostration of the system generally, has gradually recovered his health, and is now able to resume his ordinary business avocations.

I have said that the dose taken in this instance was a teaspoonful, and since we have no standard for the size of teaspoons, and choosing to be definite in this matter, I went to the store of the druggist, and filled the same teaspoon, used in administering the oil, with oil of the same quality administered—which by the way was a genuine article—and found that my patient had taken *ninety* drops of Oleum Tiglii.

BUFFALO, March 30, 1848.

